# Cheating by Exploitation of Developmental Prestalk Patterning in *Dictyostelium discoideum*

**DOI:** 10.1371/journal.pgen.1000854

**Published:** 2010-02-26

**Authors:** Anupama Khare, Gad Shaulsky

**Affiliations:** Department of Molecular and Human Genetics, Baylor College of Medicine, Houston, Texas, United States of America; Fred Hutchinson Cancer Research Center, United States of America

## Abstract

The cooperative developmental system of the social amoeba *Dictyostelium discoideum* is susceptible to exploitation by cheaters—strains that make more than their fair share of spores in chimerae. Laboratory screens in *Dictyostelium* have shown that the genetic potential for facultative cheating is high, and field surveys have shown that cheaters are abundant in nature, but the cheating mechanisms are largely unknown. Here we describe *cheater C* (*chtC*), a strong facultative cheater mutant that cheats by affecting prestalk differentiation. The *chtC* gene is developmentally regulated and its mRNA becomes stalk-enriched at the end of development. *chtC* mutants are defective in maintaining the prestalk cell fate as some of their prestalk cells transdifferentiate into prespore cells, but that defect does not affect gross developmental morphology or sporulation efficiency. In chimerae between wild-type and *chtC* mutant cells, the wild-type cells preferentially give rise to prestalk cells, and the *chtC* mutants increase their representation in the spore mass. Mixing *chtC* mutants with other cell-type proportioning mutants revealed that the cheating is directly related to the prestalk-differentiation propensity of the victim. These findings illustrate that a cheater can victimize cooperative strains by exploiting an established developmental pathway.

## Author Summary

Cooperative systems are susceptible to exploitation by cheaters who enjoy the benefits of cooperation without paying the costs. Such conflict is seen in biological systems at every level from individual genes within a cell to individuals within societies. The social amoebae *Dictyostelium discoideum* have a unique cooperative system in which large numbers of individual cells aggregate to form fruiting bodies with reproductive spores, and dead stalk cells that may help the survival and dispersal of the spores. Fruiting bodies can contain several genotypes, and hence can be exploited by cheater cells that preferentially form spores without contributing fairly to the stalk. We have studied a mutant, *cheater C* (*chtC*), which is defective in forming certain stalk cells, but is still able to form fruiting bodies on its own. However, when wild-type cells are mixed with *chtC* cells, the wild-type cells compensate for the stalk-forming defect of *chtC* and form more of the stalk cells. In that way, *chtC* cells cheat by taking advantage of developmental processes that normally regulate cell-type proportions. This study shows that existing mechanisms of developmental regulation can be exploited by cheater mutants, and the social amoebae offer a good system to study such mechanisms.

## Introduction

Cooperative behaviors are susceptible to exploitation by cheaters – individuals that do not pay the full cost of cooperation, but reap the benefits [1] and thus take advantage of other cooperative individuals (victims). Cheating is predicted to affect the relative fitness of any interacting partners, especially when multiple genotypes are involved. Such behavior is thought to occur in all cooperative societies, and has been demonstrated in several different social insect colonies [2],[3], where a significant part of the population (the workers) does not take part in reproduction. In these systems, cheaters can manipulate developmental processes, thereby changing the balance between the reproductive (queen) and supporting (worker) castes. For example, cheaters exploit cooperative genotypes by tweaking mechanisms such as the regulation of organism size [3], developmental timing [2] and differentiation into different castes [3],[4]. It is likely that the regulation of other developmental processes – cell-division and cell-fate determination, proportioning and maintenance – is also susceptible to cheating. However, it is hard to study these mechanisms at the genetic and cellular levels due to the complex nature of these social systems.

Social microorganisms are good model systems for the study of cheating mechanisms at the molecular level. The social amoebae *Dictyostelium discoideum* provide an added advantage because the cells exhibit social behavior in the context of multicellular development. *Dictyostelium* cells propagate as unicellular amoebae and feed on bacteria. However, under conditions of starvation, about 10^5^ cells aggregate and go through multicellular development. The cells give rise to a structure called the fruiting body where 70–80% of the cells form viable spores that may germinate in the next generation to form amoebae, while the remaining cells give rise to dead, vacuolated cells that contribute to stalk-formation and hence sacrifice their reproduction [5].

This developmental cycle is different from the development of metazoan organisms, since multicellularity is achieved by aggregation rather than by cell division of a fertilized egg. An important consequence is that Dictyostelids readily form organisms containing multiple clones. In such chimerae, different genotypes can contribute differently to the production of the reproductive (spores) and supporting (stalk cells) cell-types, and change their representation in subsequent generations, similar to the cheating behavior seen in insect societies. Disproportionate over-representation of a specific genotype in the spore population of a chimeric fruiting body at the cost of another strain is defined as cheating, and the over- and under-represented strains are termed as ‘cheaters’ and ‘victims’, respectively. Chimerism has been observed in nature [6], and clones isolated from the wild can cheat on one another in the laboratory [7].

The first cheater mutant identified in *D. discoideum*, *chtA* (*fbxA*), is an obligate parasite that is unable to form spores in clonal populations [8]. When mixed with *chtA*, the wild-type prespore cells differentiate into stalk cells. This is the only cheating mechanism that has been identified in *Dictyostelium* to date. However, since *chtA* does not complete development under clonal conditions, it is unlikely that its behavior is characteristic of cells in the wild since *Dictyostelium* strains are often found in clonal populations [6].

Recent studies have shown that a large number of mutations in *Dictyostelium* can lead to facultative cheating [9]. Facultative cheater mutants are capable of forming fruiting bodies in clonal populations, but cheat on wild-type cells in chimera. These mutants probably cheat by exploiting a variety of mechanisms, and the social genes identified are predicted to be involved in a variety of different cellular processes [9]. Development in *Dictyostelium* involves both the initial differentiation and proportioning of several different cell-types, and the subsequent maintenance of cell-fate and cell-type proportions. Any of these developmental mechanisms might be co-opted by selfish cheater mutants, akin to what is seen in insect societies. Consequently, the study of such cheater mutants is likely to facilitate greater understanding of specific pathways of differentiation in *Dictyostelium*, in addition to developmental cheating mechanisms in general.

We have studied *chtC* [10], one of the strongest facultative cheater mutants identified by Santorelli et al. [9]. We found that *chtC* has defects in maintaining the prestalk cell fate, and consequently is defective in the expression of certain late prestalk markers. Even though this does not lead to any discernible stalk defects when *chtC* mutants develop on their own, wild-type cells increase their prestalk differentiation in chimerae with *chtC* and are cheated upon. These findings suggest that cheaters in *Dictyostelium* can manipulate mechanisms of developmental regulation such as the maintenance of cell-type proportioning to take advantage of other strains in the population, while retaining their fitness under clonal conditions.

## Results

### The *chtC* gene

**Figure 1:**
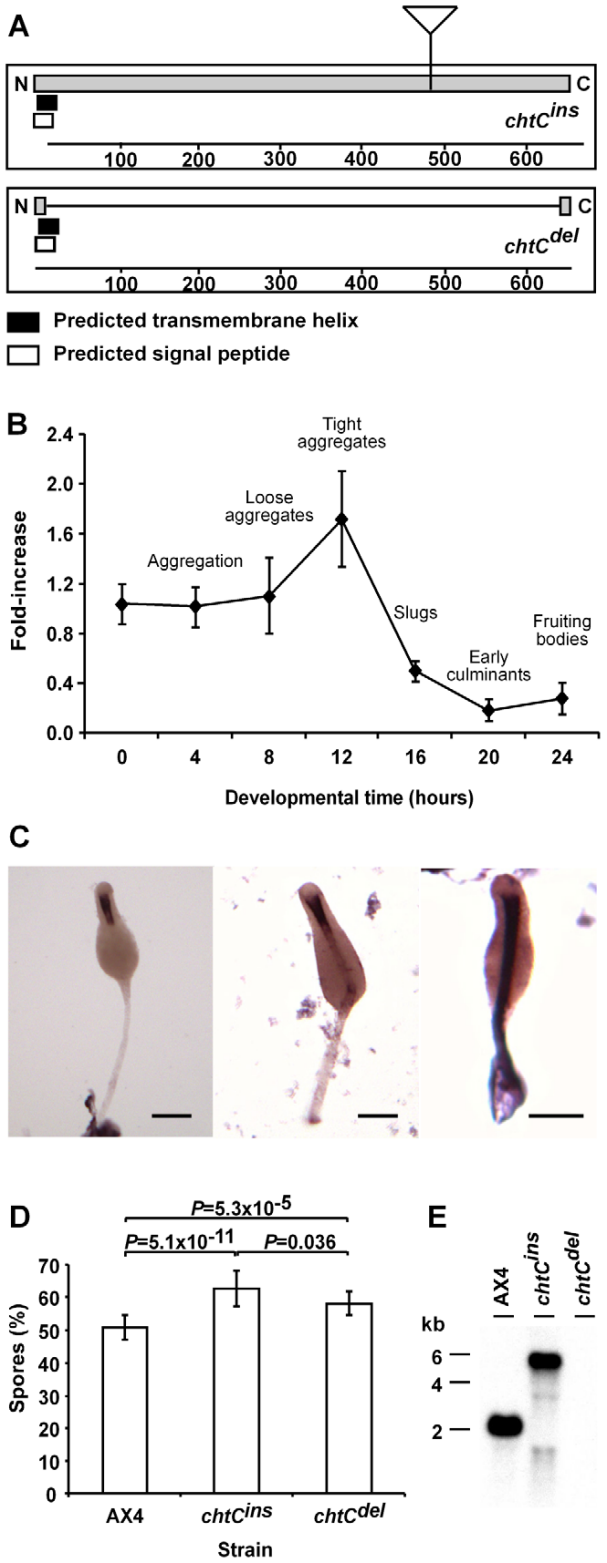
The *chtC* gene. (A) *chtC* encodes a putative transmembrane protein with a signal sequence and a single N-terminal transmembrane domain. The *chtC^ins^* mutant strain carries an insertion of the pLPBLP plasmid in the *chtC* ORF. In the *chtC^del^* mutant, most of the *chtC* ORF has been replaced by the pLPBLP plasmid. (B) Quantitative reverse-transcriptase PCR with primers specific to *chtC* performed on RNA samples collected from wild-type AX4 cells at 4-hr intervals during development as indicated on the x-axis. Data are presented as the fold change relative to the levels at 0 hrs (y-axis) and are the averages and standard errors of 3 measurements each of 2 independent biological replications. The developmental stages corresponding to the different time-points are indicated. (C) *in situ* RNA hybridization with a probe against *chtC* on whole-mount late culminant structures (22–24 hours of development). Staining is enriched in the stalk and specifically in the funnel (the upper part of the stalk). The scale bar represents 0.1 mm. (D) Spore production of the wild type (AX4) and the two mutants (*chtC^ins^* and *chtC^del^*) when mixed in a 1∶1 ratio with AX4-GFP cells and developed as chimerae. The data are presented as the proportion (%) of the spores produced by the strain of interest relative to the total spores produced by the chimerae. The results are the means and standard errors of at least 8 independent replications. The *chtC* mutants form significantly more spores compared to AX4 (Student's *t*-test) and the *chtC^ins^* mutant cheats significantly more than the *chtC^del^* mutant (Student's *t*-test). The *P*-values for each pair (corrected for multiple testing using the ‘Benjamini and Hochberg’ method) are shown above the respective bars. (E) Northern blot analysis, with a probe against *chtC*, of total RNA prepared from 8-hr cells. The genotypes are indicated above the lanes and the molecular weights (kilobase) are indicated on the left.

LAS5 was one of the strongest cheater strains identified in a large scale screen for cheater mutants [9]. This mutant strain has a plasmid insertion in the *chtC* gene [10]. The *chtC* gene is predicted to encode an approximately 75 kDa protein with a signal peptide anchor and a transmembrane domain at the N-terminus (Figure 1A). This protein has orthologs of unknown function with about 20% identity in ciliates such as *Paramecium tetraurelia* and *Tetrahymena thermophila*, but no detectable homology to proteins in other organisms (data not shown). The gene is also up-regulated in AX2 cells incubated with *E. coli* when compared to cells incubated in axenic medium [11]. To determine the expression properties of the *chtC* transcript, we collected RNA from AX4 cells at 4-hour intervals throughout development and performed quantitative reverse transcription PCR (Q-RT-PCR) with *chtC*-specific primers (Figure 1B). We found *chtC* mRNA at all times with a peak at 12 hours of development, when the cells were at the tight aggregate stage, followed by a decline at 16 hours and comparatively lower levels thereafter. We also tested the spatial expression pattern of *chtC* by whole mount *in situ* RNA hybridization. The *chtC* mRNA was uniformly abundant in all cells during the finger stage of development (data not shown), but became highly enriched in the stalk, with the highest levels in the funnel (Figure 1C), which is at the top of the stalk tube, during late culmination (fruiting body formation).

### Different alleles of *chtC* lead to cheating

The original mutant, LAS5, had a pBSR1 plasmid insertion at nucleotide 1377 of the *chtC* ORF (Figure 1A). We generated two new alleles of chtC. The *chtC^ins^* mutant contains a plasmid insertion at position 1377 of the endogenous locus and the *chtC^del^* mutant contains a plasmid instead of the endogenous region that codes for amino acid 13 – 642 (Figure 1A). Both strains were made sensitive to Blasticidin S to facilitate the analysis of chimerae. The alleles were confirmed by Southern blot analysis and by PCR across the relevant insertion sites (data not shown).

We first tested the spore-forming ability of the *chtC* mutants. Sporulation of the clonal *chtC* mutants and of 1∶1 chimerae between the *chtC* mutants and AX4, were indistinguishable from that of clonal AX4 populations, as tested by determining spore morphology (data not shown), sporulation efficiency, resistance to detergent, and germination efficiency (Figure S1). This finding is in contrast to the original LAS5 mutant which had a higher sporulation efficiency compared to AX4 cells [9], suggesting that different alleles of *chtC* can lead to distinct phenotypes. We then studied the behavior of the *chtC* mutants in chimera. We first tested whether the *chtC* mutants co-aggregate with wild-type cells by observing 1∶1 mixtures of either *chtC^ins^* or *chtC^del^* with AX4 at 8 hours of development (Figure S2). Both the *chtC* mutants co-aggregated with AX4 cells, similar to an AX4 control. We then tested the cheating behavior of the *chtC* mutants by mixing either *chtC^ins^* or *chtC^del^* at a 1∶1 ratio with AX4/[*act15*]:GFP (AX4-GFP) cells and letting the mixed populations complete development to form fruiting bodies. We also mixed AX4-GFP cells with unlabeled AX4 cells as a control. Following development, we collected all the cells, selected for spores by detergent treatment, and counted the ratio of fluorescent to non-fluorescent spores. In the control mixes we found that the AX4 cells form approximately 50% of the spores, suggesting that the AX4-GFP strain behaves in an almost identical fashion to AX4 (Figure 1D). Both the *chtC^ins^* and the *chtC^del^* mutants cheated - they formed a significantly higher number of spores than AX4 (Figure 1D). Further, the *chtC^ins^* mutant cheated significantly more than the *chtC^del^* mutant did, suggesting that the *chtC^ins^* mutant is not a null mutant. We then tested the two mutants by developing them in a 1∶1 mixture with each other. The *chtC^ins^* mutant cheated on the *chtC^del^* mutant by forming 60.7%±5.7% spores, which is significantly greater than the hypothesized value of 50% (n = 3, one-sample one-sided *t*-test, *P* = 0.041).

Thus the *chtC^ins^* mutant is distinct from the *chtC^del^* mutant, suggesting that it is not a null, but possibly a gain-of-function allele. In order to test this possibility further, we performed Northern blot analysis with a *chtC* probe on 8-hour RNA samples from AX4 and from the *chtC^ins^* and *chtC^del^* mutants (Figure 1E). The wild type *chtC* transcript size is 2 kb, as expected from the predicted gene model. The *chtC^del^* mutant does not express detectable levels of the transcript, consistent with the deletion of nearly the entire gene and confirming the hypothesis that it is a null-mutant. The *chtC^ins^* strain expresses a 5–6 kb transcript. Northern blot analysis with a probe against the inserted plasmid showed that this was due to read-through transcription into the plasmid insertion (data not shown). We also performed RT-PCR with primers against the region of the *chtC* gene downstream of the insertion and observed a product (data not shown). These data suggest that the *chtC^ins^* mutant expresses an aberrant transcript that extends across the inserted plasmid and back into the *chtC* gene.

### The *chtC* mutants are defective in maintaining the prestalk cell fate

The cheating behavior of the mutant strains and the stalk-enriched expression of the *chtC* mRNA during late developmental stages suggested that *chtC* may play a role in stalk development although the ubiquitous expression of the gene at earlier stages may imply a role in prespore cells or spores as well. Nevertheless, the *chtC* mutant strains appear morphologically indistinguishable from the parental AX4 strain during growth and development, (Figure S1 and data not shown). We therefore tested other stalk phenotypes of the *chtC* mutants. During development of wild-type *D discoideum*, the small molecule DIF-1 (Differentiation Inducing Factor-1) induces the differentiation of stalk cells, and inhibits spore-differentiation, and sensitivity to this molecule is important for the differentiation of a specific sub-type of prestalk cells. After differentiation, prestalk cells are localized in the anterior part of a developing slug, where they are required for proper slug migration. Finally, wild-type fruiting bodies in *D. discoideum* contain stalks that consist of vacuolated cells and cellulose deposits, which are important for the formation of a properly structured stalk [5]. We tested each of these stalk phenotypes in the *chtC* mutants by examining squashes of culminants (fruiting bodies) using high-power phase-contrast microscopy, staining for cellulose with the fluorescent dye calcofluor [12], testing for DIF-1 sensitivity by the cAMP-removal and 8-Br-cAMP monolayer assays [13], and testing slug migration. We found no significant difference between AX4 and the *chtC* mutants in these assays (data not shown).

**Figure 2:**
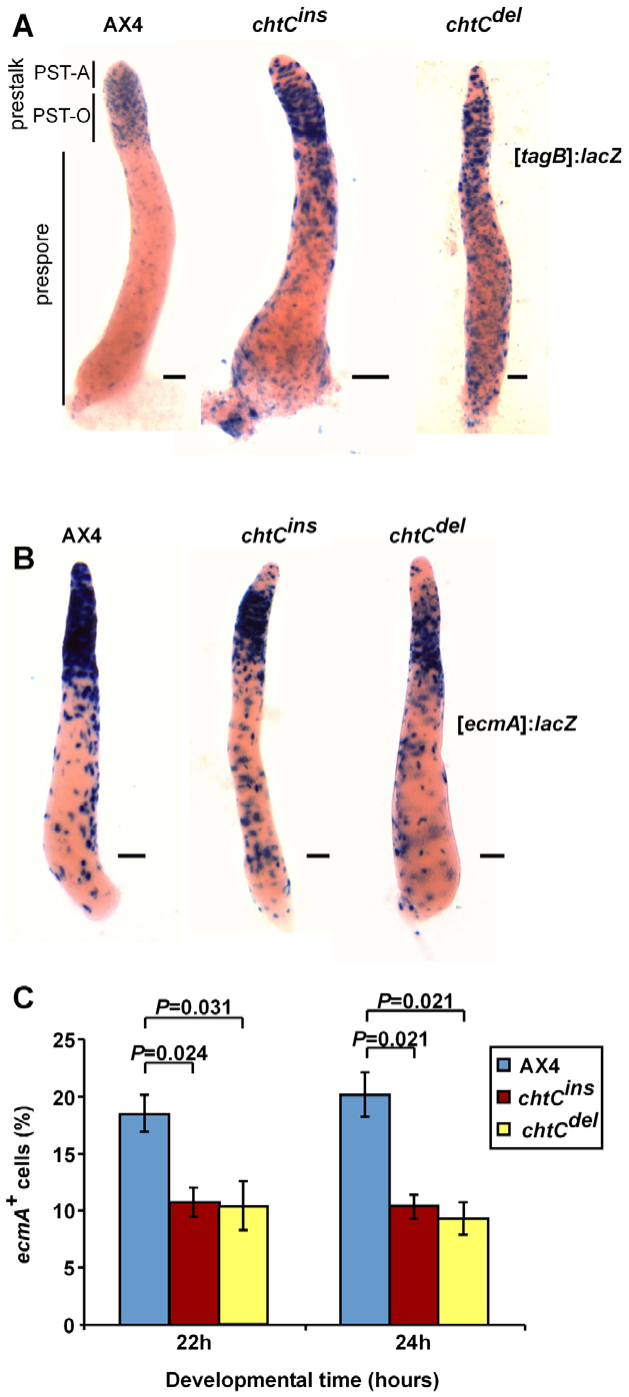
The *chtC* mutants exhibit prestalk defects. AX4, *chtC^ins^* and *chtC^del^* strains labeled with either [*tagB*]:*lacZ* (A) or [*ecmA*]:*lacZ* (B) were developed for 16 hours, fixed and stained with X-gal. In both cases, 20% of the cells were labeled and the remaining population consisted of the unlabeled parental strain. Representative slugs for each strain are shown. The scale bars represent 0.1 mm. The different parts of the slug are shown in (A). (C) Multicellular structures of AX4, *chtC^ins^* and *chtC^del^* were dissociated after 22 and 24 hours of development, fixed and stained with X-gal, and the number of blue cells was determined. The data are shown as the proportion (%) of stained cells relative to the entire population. The results are the means and standard errors of at least 3 independent replications. Brackets above the respective bars indicate that the *chtC^ins^* and the *chtC^del^* mutants have significantly fewer stained cells as compared to AX4 (Student's *t*-test). Individual *P*-values (corrected for multiple testing by the ‘Benjamini and Hochberg’ method) are indicated above the bars.

To study stalk differentiation in greater detail, we used 2 different prestalk markers – *tagB* and *ecmA*. Expression of the *tagB* gene is induced 8 hours into development in prestalk cells, about 4 hours earlier than *ecmA* [14]. Also, unlike *ecmA*, expression of the *tagB* gene is not induced by DIF-1 [15]. We developed [*tagB*]:*lacZ* labeled strains of both the *chtC^ins^* and *chtC^del^* mutants, and stained for β-galactosidase activity. In AX4 cells, *tagB* is expressed in the entire prestalk region [14]. Both the *chtC^ins^* and *chtC^del^* mutants showed strong staining in the posterior half of the prestalk region (the prestalk-O or PST-O region [16]), and weaker staining in the anterior half (the prestalk-A or PST-A region). There was also significant staining in the prespore region, suggesting that some prespore cells express the *tagB* marker or have expressed it prior to becoming prespore cells (Figure 2A). In order to test this possibility, we examined the spores made by *chtC* mutants labeled with the [*tagB*]:*lacZ* marker. We found a 100-fold increase in the proportion of *tagB*-positive spores formed by either of the *chtC* mutants, compared to AX4 (Table 1).

**Table 1:** *chtC* spores have a prestalk history.

Sample	% *lacZ* ^+^ spores	SEM
**Filter development**		
50% AX4/[*tagB*]:*lacZ* +50% AX4	<0.01%	
50% *chtC^ins^*/[*tagB*]:*lacZ* +50% *chtC^ins^*	1.2%	0.3%
50% *chtC^ins^*/[*tagB*]:*lacZ* +50% AX4	2.0%	0.4%
50% *chtC^del^*/[*tagB*]:*lacZ* +50% *chtC^del^*	2.1%	0.3%
50% *chtC^del^*/[*tagB*]:*lacZ* +50% AX4	1.7%	0.5%
**Post slug-migration**		
AX4/[*tagB*]:*lacZ*	<0.1%	
*chtC^ins^*/[*tagB*]:*lacZ*	8.9%	1.2%
*chtC^del^*/[*tagB*]:*lacZ*	12.9%	2.1%

The deficit of [*tagB*]:*lacZ*-expressing cells in the PST-A region, combined with the increase in prespore cells that express [*tagB*]:*lacZ* suggests that the *tagB-*expressing prestalk cells, which contribute to the PST-A region in the wild type, are undergoing transdifferentiation and form spores instead of stalk cells. An increase in this transdifferentiation in the presence of AX4 cells would be a potential mechanism of cheating. However, we observed no significant change in the proportion of [*tagB*]:*lacZ*-positive spores when the *chtC* mutants were mixed with unlabeled AX4 instead of the unlabeled *chtC* mutant cells (Table 1).

Prolonged migration of *Dictyostelium* slugs results in increased transdifferentiation of prestalk cells into spores [17],[18]. To test whether the *chtC* mutants showed increased transdifferentiation under such conditions, we allowed the [*tagB*]:*lacZ* labeled *chtC* mutants to migrate for 48 hours, and then induced culmination. We collected spores, stained them with X-gal, and counted the number of stained spores (Table 1). In the *chtC* mutant strains, 8–12% of the spores were labeled, suggesting that they had a prestalk history. Thus, a significant proportion of the *chtC* mutant population undergoes a cell-fate transformation, suggesting that the *chtC* gene is required for the maintenance of the prestalk cell fate.

In order to further dissect the prestalk properties of the *chtC* mutants, we generated *chtC* mutant strains expressing *lacZ* under the prestalk promoter, *ecmA*. We developed these strains, and stained for β-galactosidase activity. Both the *chtC^ins^* and *chtC^del^* mutants showed strong staining in the PST-O region, but weaker staining in the PST-A region (Figure 2B), similar to the phenotype seen in the [*tagB*]:*lacZ* strains, suggesting that in the *chtC* mutants, the cells in the PST-A region are defective in both *tagB* as well as *ecmA* expression. However, there was no discernible change in the expression of *ecmA* in the prespore region, compared to AX4. We quantified this phenotype by dissociating the structures during late culmination and counting the number of cells that stained positively for β-galactosidase activity. Both the *chtC* mutants formed significantly fewer *ecmA* positive cells than AX4 (Figure 2C). There was no significant change in the proportion of *ecmA* positive cells when the labeled *chtC* strains were mixed with either the unlabeled parent or unlabeled AX4 (data not shown).

To determine the timing of transdifferentiation, we determined the proportion of *ecmA*-positive spores formed by the *chtC* mutants using the [*ecmA*]:*lacZ* labeled strains. We found no significant difference compared to AX4 [*ecmA*]:*lacZ* cells (data not shown). This finding suggests that in the *chtC* mutants, a population of prestalk cells that would otherwise have given rise to PST-A cells changes its cell fate and goes on to form spores instead. This process takes place soon after the initial prestalk-cell differentiation - after the induction of *tagB* expression, but before *ecmA* induction, a timing that coincides with the peak in *chtC* mRNA levels (Figure 1B). We further investigated this process by comparing *tagB* expression levels in 16 h slugs and in fully differentiated spores in both the *chtC* mutants and in the parental wild type cells (Figure S3). The level of *tagB* mRNA was significantly lower in the spores at 24 h as compared to the level in slugs at 16 h, suggesting that the *tagB* expression observed in the spores of the *chtC* mutants is not due to a wholesale induction of *tagB* expression in prespore cells but rather to a transdifferentiation of a small proportion of the prestalk cells. Even though the *chtC^ins^* mutant had higher levels of *tagB* expression at 16 h of development (compared to AX4), the level of *tagB* mRNA in the spores for both the *chtC* mutants was not significantly increased compared to a similar AX4 control. These data further support the conclusion that the blue staining observed in spores of the [*tagB*]:*lacZ* labeled *chtC*-mutants reflects transdifferentiation of prestalk cells into prespore cells.

Interestingly, even though the PST-A region in the *chtC* mutant slugs is defective for the expression of two separate markers – *tagB* and *ecmA* – the *chtC* mutants have no overt defects in stalk morphology or function, suggesting that under laboratory conditions, the expression of these markers is not required for proper PST-A cell function.

We also tested whether the *chtC* gene was required to maintain the prespore cell fate, by observing slugs of either AX4, *chtC^ins^* or *chtC^del^* expressing the [*cotB*]:*lacZ* marker (*cotB* is a well-established prespore marker that is expressed exclusively in prespore cells and spores) [19]. Neither mutant strain expressed the *cotB* marker in the prestalk region (Figure S4), suggesting that the *chtC* mutant cells are not undergoing transdifferentiation from prespore to prestalk cells and that the directional transdifferentiation we observe is not due to a general defect in cell type differentiation.

### The *chtC* mutants increase prestalk differentiation of wild-type cells in chimerae

**Figure 3:**
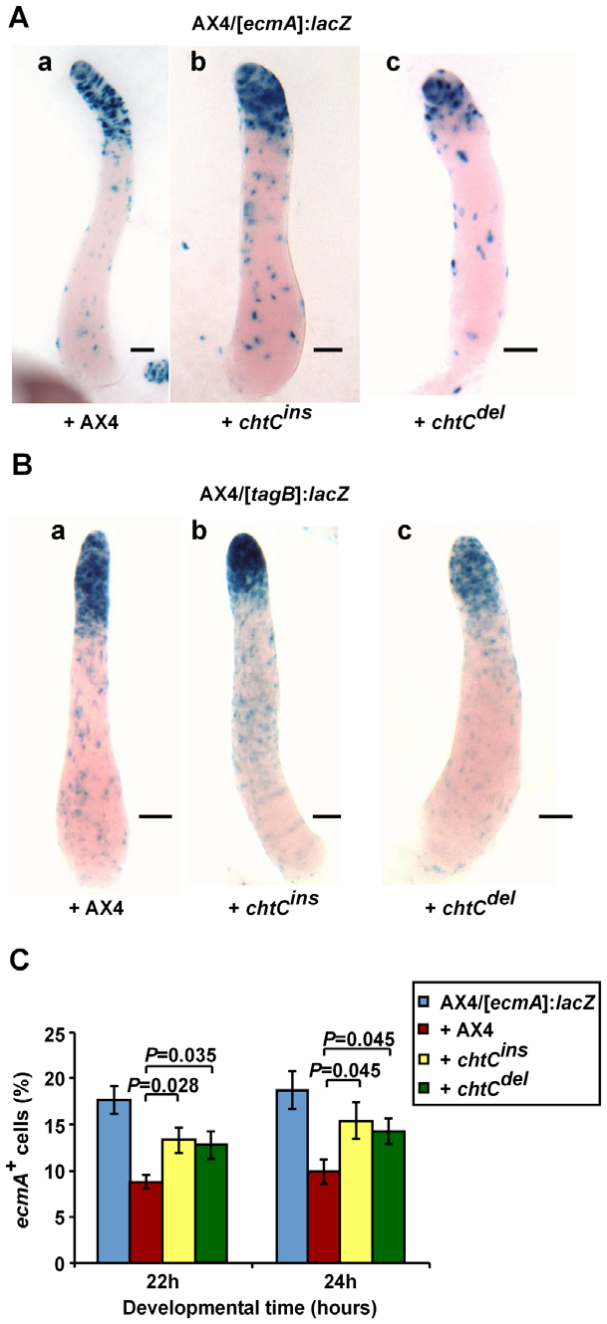
The *chtC* mutants affect prestalk development of AX4 in chimerae. Strains were grown clonally and then mixed at the appropriate proportions and developed in chimerae. AX4 cells labeled with either [*ecmA*]:*lacZ* (A) or [*tagB*]:*lacZ* (B) were mixed in a 1∶4 ratio with unlabeled AX4, *chtC^ins^* or *chtC^del^* cells as indicated. Multicellular structures were fixed and stained with X-gal after 16 hours of development. Representative slugs for each chimeric mixture are shown. The scale bars represent 0.1 mm. (C) AX4/[*ecmA*]:*lacZ* cells were developed either clonally, or in a 1∶1 mix with unlabeled AX4, *chtC^ins^* or *chtC^del^* cells. Multicellular structures at 22 h and 24 h of development were dissociated, the cells were stained with X-gal and the number of blue cells was determined. The data are shown as the proportion (%) of stained cells relative to the entire population. The results are the means and standard errors of 6 independent replications. The number of stained AX4 prestalk cells is significantly increased in the presence of the *chtC* mutants, compared to AX4 (Student's *t*-test). Individual *P*-values (corrected for multiple testing by the ‘Benjamini and Hochberg’ method) are indicated above the bars.

The *chtC* mutants are defective in the maintenance of the prestalk cell-fate. We hypothesized that this defect in *chtC* cells would affect prestalk differentiation of AX4 cells in chimera. In order to test this hypothesis, we examined the pattern of AX4 prestalk cells in chimeric populations. We developed mixed populations of 20% AX4/[*ecmA*]:*lacZ* cells and 80% unlabeled *chtC* cells. When mixed with either the *chtC^ins^* or the *chtC^del^* mutant, the AX4/[*ecmA*]:*lacZ* cells were preferentially localized in the PST-A region (Figure 3A). We repeated the experiment using the AX4/[*tagB*]:*lacZ* strain [15], and found similar results (Figure 3B). These experiments were also carried out at a 1∶1 ratio between AX4 cells and the *chtC* mutants, and qualitatively similar results were observed (data not shown), though the effects were more pronounced at a 1∶4 ratio.

To quantify this finding, we mixed AX4/[*ecmA*]:*lacZ* cells with each of the *chtC* mutants, and developed them in chimera. We collected samples at 22 and 24 hours, dissociated the structures and counted the number of cells that stained positive for β-galactosidase activity. The presence of either of the two *chtC* mutants caused an increase in the number of *ecmA* positive cells in AX4 (Figure 3C), suggesting that the *chtC* mutants may cheat by causing an increase in the proportion of AX4 prestalk cells.

A simple explanation of these results is that in chimera, a defect in prestalk differentiation in the PST-A region of the *chtC* mutants is compensated for by AX4 cells, which then occupy the PST-A region to fill the void, and differentiate into more prestalk cells. As such chimeric mixtures complete development, AX4 cells thus form a smaller proportion of spores compared to the *chtC* mutants, and get cheated upon. In clonal *chtC* populations, in spite of the defective prestalk marker expression, cells of the *chtC* mutants take on the PST-A cell-fate and are able to form morphologically normal fruiting bodies, with similar numbers of spores compared to clonal AX4 populations.

### The *chtC* mutants have divergent effects on other mutants that affect prestalk cells

The model proposed above predicts that the ability of the victim to contribute to the PST-A region is important for the cheating mechanism of the *chtC* mutants. If the model were correct, the cheating phenotype of the *chtC* mutants would be correlated with the ability of their chimeric counterparts to contribute to the PST-A region, and consequently differentiate an increased number of prestalk cells. In order to test this prediction, we mixed the *chtC* mutants with two other mutants that avoid the PST-A region in chimera with AX4 cells, the *tagA^–^* and *tagB^–^* mutants, and examined prestalk differentiation and spore production.

### The *tagA^–^* mutant

**Figure 4:**
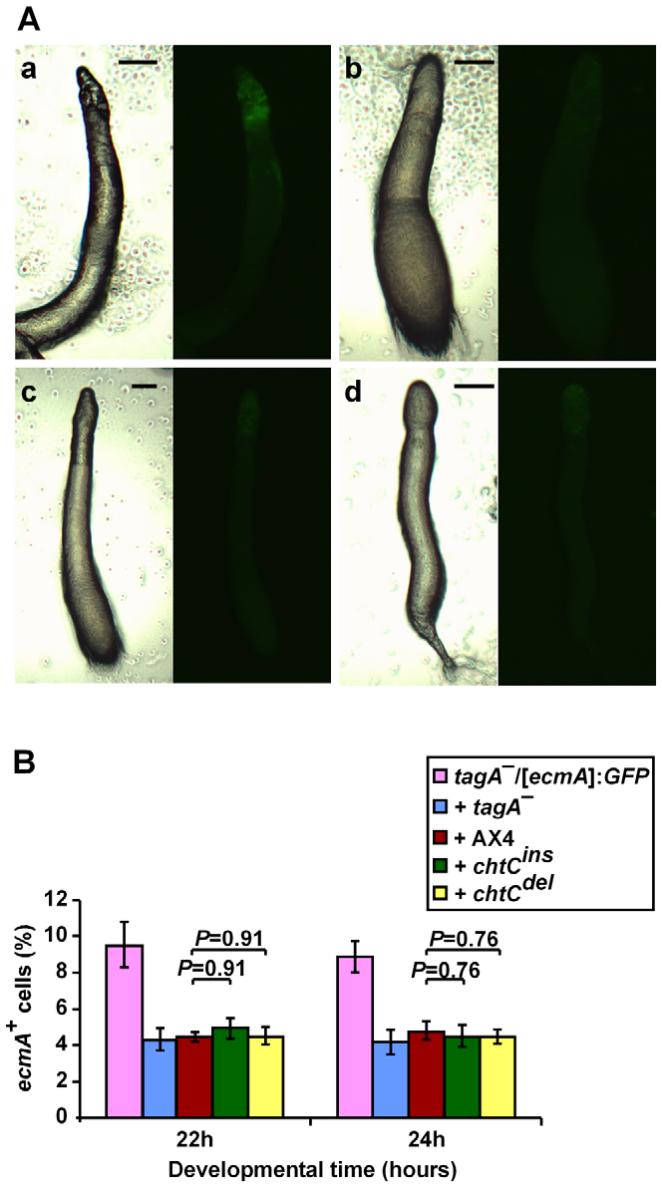
The *tagA^–^* mutant is unaffected by the presence of *chtC* mutants in chimerae. The strains were grown separately and mixed in the appropriate proportions before development in chimera. (A) We photographed multicellular structures after 16 hours of development under phase-contrast microscopy (left panels) and fluorescence microscopy (right panels). The *tagA^–^*/[*ecmA*]:*GFP* strain was mixed in a 1∶1 ratio with unlabeled *tagA^–^* cells (a), unlabeled AX4 cells (b), unlabeled *chtC^ins^* cells (c), and unlabeled *chtC^del^* cells (d). The entire prestalk region (shown by white arrows) is fluorescently labeled in a, but very little fluorescence in seen in b-d. Representative slugs for each chimeric mixture are shown. The scale bars represent 0.1 mm. (B) The *tagA^–^*/[*ecmA*]:*GFP* strain was developed either clonally or in a 1∶1 mix with unlabeled *tagA^–^*, AX4, *chtC^ins^*, or *chtC^del^* cells. We disaggregated the cells after 22 h and 24 h of development, and determined the proportion of GFP-positive cells by counting under the fluorescence microscope. The data are shown as the proportion (%) of fluorescent cells relative to the entire population. The results are the means and standard errors of 3 independent replications. The number of prestalk cells formed by the *tagA^–^* mutant is not significantly different in the presence of the *chtC* mutants, as compared to AX4 (Student's *t*-test). Individual *P*-values (corrected for multiple testing by the ‘Benjamini and Hochberg’ method) are indicated above the bars.

The *tagA^–^* and the *tagA^–^*/[*ecmA*]:*GFP* strains were described previously [20],[21]. The *tagA^–^* mutant has defects in cell-type specification, and does not contribute to the PST-A region and to the terminal stalk structure in chimera with AX4 cells. We examined the patterning of the *tagA^–^*/[*ecmA*]:*GFP* cells at the slug stage of development. As expected, the *tagA^–^*/[*ecmA*]:*GFP* cells showed a wild-type like pattern of fluorescence in the anterior part of the slug when developed as a clonal population (data not shown) and in 1∶1 mixtures with the unmarked *tagA^–^* strain (Figure 4A a). In chimerae with AX4, the *tagA^–^*/[*ecmA*]:*GFP* cells showed almost no fluorescence in the prestalk region, consistent with the published observations [20] (Figure 4A b). The results of mixing the *tagA^–^*/[*ecmA*]:*GFP* cells with either one of the *chtC* mutants were nearly indistinguishable from that seen when mixing *tagA^–^*/[*ecmA*]:*GFP* with the wild type (Figure 4A c,d).

Since the *tagA^–^* prestalk cells do not appear to occupy the PST-A region in chimera with the *chtC* mutants, we predicted that the proportion of *tagA^–^* prestalk cells would also be unaffected in chimerae with *chtC*. To test this prediction, we mixed *tagA^–^*/[*ecmA*]:*GFP* cells with either of the *chtC* mutants in a 1∶1 ratio, developed them and counted the proportion of fluorescently labeled cells after 22 and 24 hours of development. Neither the *chtC^ins^* nor the *chtC^del^* mutant affected the proportion of [*ecmA*]:*GFP* positive cells formed by the *tagA^–^* mutant (Figure 4B). Thus the *tagA^–^* mutant appears unaffected by the presence of the *chtC* mutants in chimera, unlike the phenotype seen in the case of wild-type cells (though it is possible that the lower sensitivity of detection of the GFP reporter as compared to β-galactosidase may be preventing the observation of subtle effects). According to our model, these data would suggest that the *chtC* mutants should not be able to cheat on the *tagA^–^* mutant.

### The *tagB^–^* mutant

**Figure 5:**
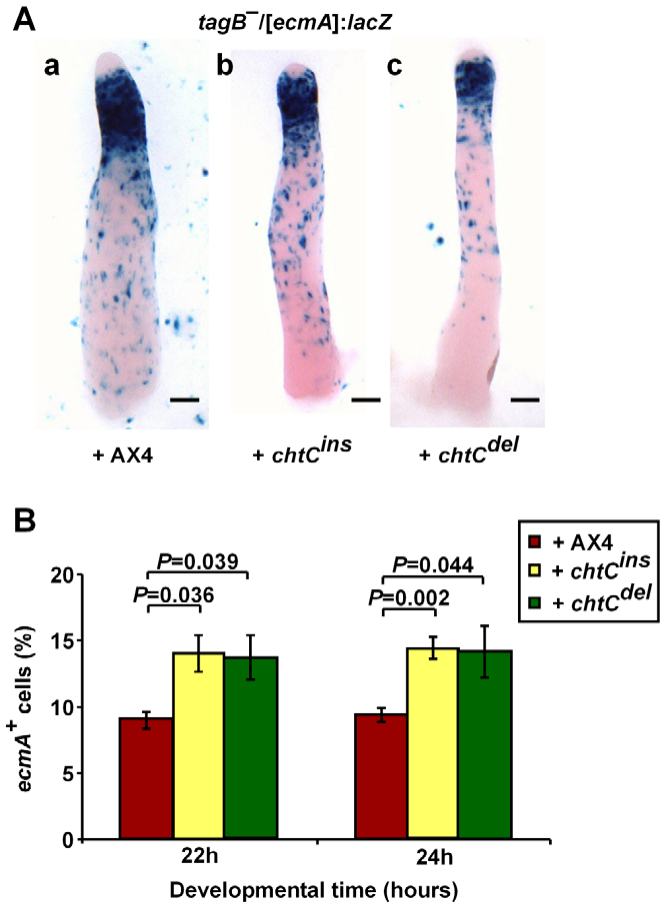
The *tagB^–^* mutant behaves like AX4 in chimerae with the *chtC* mutants. The strains were grown separately and mixed in the appropriate proportions before development in chimera. (A) Developing structures were fixed and stained after 16 h of development. The *tagB^–^*/[*ecmA*]:*lacZ* strain was mixed in a 1∶4 ratio with unlabeled AX4 cells (a), unlabeled *chtC^ins^* cells (b), and unlabeled *chtC^del^* cells (c). Representative slugs for each chimeric mixture are shown. The scale bars represent 0.1 mm. (B) The *tagB^–^*/[*ecmA*]:*lacZ* strain was developed either clonally or in a 1∶1 mix with unlabeled AX4, *chtC^ins^* or *chtC^del^* cells. We disaggregated the cells after 22 or 24 h of development, stained with X-gal and determined the number of blue cells. The data are shown as the proportion (%) of stained cells relative to the entire population. The results are the means and standard errors of at least 4 independent replications. The number of stained prestalk cells formed by the *tagB^–^* mutant is significantly increased in the presence of the *chtC* mutants, as compared to AX4 (Student's *t*-test). Individual *P*-values (corrected for multiple testing by the ‘Benjamini and Hochberg’ method) are indicated above the bars.

We performed similar experiments with the *tagB^–^* and *tagB^–^*/[*ecmA*]*:lacZ* strains [14]. The *tagB^–^* mutant is unable to proceed beyond the tight aggregate stage of development in a clonal population. However, in chimera with AX4, *tagB^–^* cells can proceed through development, but do not contribute to the PST-A region or to the stalk. We studied the patterning of the *tagB^–^*/[*ecmA*]:*lacZ* cells at the slug stage of development. As expected, the *tagB^–^*/[*ecmA*]:*lacZ* cells occupy the PST-O zone when mixed with AX4 cells at a 1∶4 ratio, leaving a substantial portion of the tip (PST-A region) unstained (Figure 5A a). However, in 1∶4 chimerae with the *chtC* mutants, the *tagB^–^* prestalk cells were considerably anteriorized, and occupied a larger portion of the PST-A zone (Figure 5A b,c). Similar results were seen at a 1∶1 ratio between the *tagB^–^* cells and the *chtC*-mutants (data not shown), though the phenotype was more pronounced in the 1∶4 chimerae. Based on this observation, our model predicts that the *tagB^–^* mutant would differentiate more prestalk cells in chimerae with the *chtC* mutants. We tested this prediction and observed that in chimerae with the *chtC* mutants, the *tagB^–^*/[*ecmA*]:*lacZ* strain produced a higher proportion of [*ecmA*]:*lacZ* positive prestalk cells (Figure 5B), similar to the phenotype seen when AX4 is mixed with the *chtC* mutants.

Thus, the *tagB^–^* mutant cells behave like the wild type AX4 cells in chimerae with the *chtC* mutants, suggesting that the *chtC* mutants would cheat on *tagB^–^* cells.

### Cheating by the *chtC* mutants is correlated with the effect on prestalk differentiation

**Figure 6:**
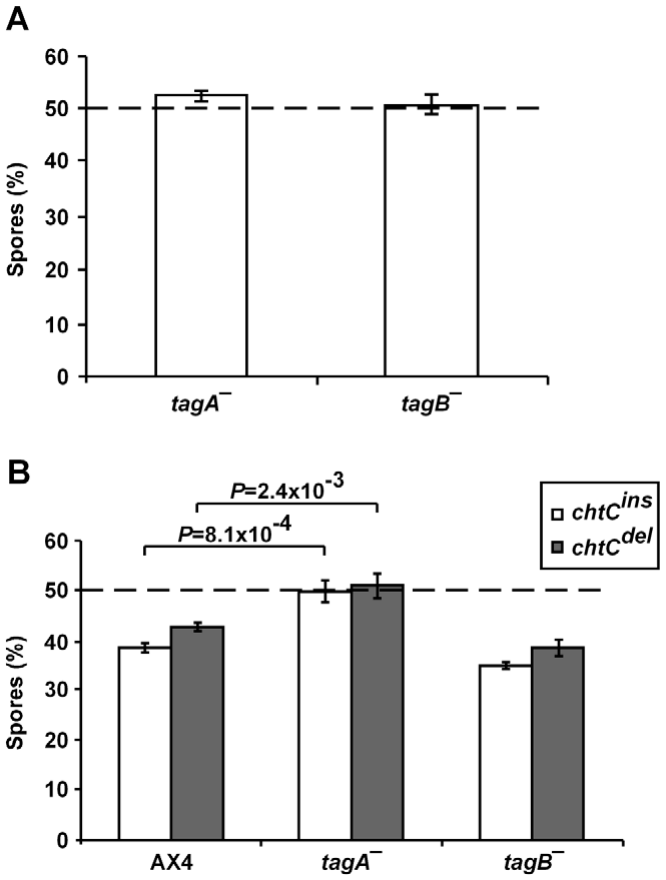
The *chtC* mutants cheat on *tagB^–^*, but not on *tagA^–^*. The strains were grown separately and mixed in the appropriate proportions before development in chimera. (A) Spore production of the *tagA^–^* and *tagB* ^–^ mutants when mixed in a 1∶1 ratio with AX4-GFP cells. The data are presented as the proportion (%) of the spores produced by the strain of interest relative to the total spores produced by the chimerae. The results are the means and standard errors of at least 3 independent replications. Both the *tagA^–^* and *tagB* ^–^ mutants form approximately 50% of the spores, showing that they are neutral in terms of cheating behavior. (B) Spore production of AX4-GFP, *tagA^–^* and *tagB* ^–^ when mixed in a 1∶1 ratio with *chtC^ins^* and *chtC^del^* cells. The data are presented as the proportion (%) of the spores produced by the strain of interest relative to the total spores produced by the chimerae. The results are the means and standard errors of at least 3 independent replications. In the chimerae with either *chtC* mutant, the *tagA^–^* mutant forms significantly more spores and the *tagB^–^* mutant is not significantly different, compared to AX4 (Student's *t*-test). Individual *P*-values are indicated above the bars for the significantly different strains.

We first tested the spore production of the *tagA^–^* and *tagB^–^* mutants in control chimerae with the wild type AX4. We grew the strains clonally, mixed each strain at a 1∶1 ratio with AX4 cells and allowed the chimerae to develop. We determined the ratio of spores formed by each strain after development (Figure 6A). In terms of cheating, both the *tagA^–^* and the *tagB^–^* mutants were neutral when compared to AX4, each forming approximately 50% of the spores in the 1∶1 mix.

We then performed mixing experiments between the *chtC* mutants and either the *tagA^–^* or the *tagB^–^* mutants (Figure 6B). We found that neither *chtC^ins^* nor *chtC^del^* cheated on the *tagA^–^* mutant, but both cheated on the *tagB^–^* mutant. These results correlate well with the effects of the *chtC* mutants on the prestalk differentiation of the *tagA^–^*and the *tagB^–^* mutants in chimerae with *chtC*, thus supporting our hypothesis.

## Discussion

This study describes the first analysis of a facultative cheater mutant in *Dictyostelium*. Mutants that lack *chtC* gene function sporulate normally in clonal populations, but cheat on wild-type cells in chimerae. The two mutant alleles we have generated, *chtC^ins^* and *chtC^del^*, share most but not all of their cheating phenotypes. The *chtC^ins^* mutant is a “stronger” cheater since it cheats at a higher proportion on AX4, and cheats on the *chtC^del^* mutant. It is also a slightly better cheater when mixed with either *tagA^–^* or *tagB^–^*. The *chtC^del^* allele is a loss-of-function allele, by definition. Therefore, based on the phenotypic differences between the two mutant alleles, and due to the expression of a fusion transcript in the *chtC^ins^* mutant, we propose that the insertion generated a gain-of-function allele. One possibility is that the neomorphic *chtC^ins^* allele impairs the functioning of other proteins in the pathway, via aberrant interactions. However, our data do not provide any molecular insight into this phenomenon.

### The *chtC* gene is required for maintenance of the prestalk cell fate

The *chtC* mutants undergo a transformation of cell-fate, since cells with a prestalk history form spores. This is coincident with a PST-A specific defect in the expression of prestalk markers such as *tagB* and *ecmA*, suggesting that cells fated to occupy the PST-A region transdifferentiate and form spores instead. Thus the *chtC* gene appears to be involved in the maintenance of the PST-A cell-fate. This idea is also supported by the cell-type specificity of *chtC* gene expression, since during late development, *chtC* is the most stalk-enriched gene described to date, being expressed predominantly in the stalk, and not in other prestalk-derived tissues like the upper and lower cups. Thus, *chtC* is one of the few genes identified to be involved in maintaining cell-fate [21]–[23]. It is interesting to note that despite the defects in maintenance of the prestalk cell-fate and expression of prestalk markers, stalk morphology and function in the *chtC* mutants appears indistinguishable from that of the wild type.

This finding raises the question of why the *chtC* mutants have not spread within the population, and why the *chtC* gene still exists in the genome in *Dictyostelium*. It is possible that the *chtC* mutants have fitness defects in growth or development in the wild, or under specific environmental conditions that we have not explored in the laboratory. Additionally, it has been shown that mutants that can resist cheating by the *chtC^ins^* mutant can be selected for in a population containing the *chtC^ins^* mutant [10]. Such cheater-resistors can even inhibit the cheating by the *chtC* mutants, and may thus help to maintain the *chtC* gene in the population [10].

### Void in prestalk differentiation in the *chtC* mutants likely leads to cheating

The *chtC* mutants differentiate a population of cells that express prestalk markers, but adopt the prespore cell-fate. This transdifferentiation is associated with a decrease in the number of cells that express the late prestalk marker *ecmA*. In chimerae between AX4 and *chtC* cells, the AX4 cells differentiate a higher number of *ecmA*-positive cells. The simplest explanation for these observations is that the void in prestalk cells in *chtC* is detected by the AX4 cells, which then compensate by differentiating more prestalk cells.

The proportions between prestalk and prespore cells are almost constant in *Dictyostelium* slugs over a wide range of total cell numbers, indicating that well-regulated proportioning mechanisms control the initial differentiation of prestalk and prespore cells [24]. Our data support the hypothesis that there is a feedback mechanism that helps to sense the proportions of properly differentiated prestalk cells, and regulates the differentiation of as yet undifferentiated cells into the required cell-types as development proceeds.

### Prestalk patterning is important for cheating by the *chtC* mutants

The presence of the *chtC* mutants in chimerae affects the prestalk differentiation and patterning of the wild-type cells, which is likely to be the direct mechanism of cheating. In order to test whether prestalk patterning was important for cheating, we utilized two other prestalk differentiation mutants - *tagA^–^* and *tagB^–^*. In both cases, the ability of the *chtC* cells to affect patterning was directly correlated to the cheating behavior, suggesting that the patterning was indeed important for cheating. Nevertheless, the ability to cause wild-type cells to occupy the PST-A zone in chimera does not necessarily equate to cheating, since neither *tagA^–^* nor *tagB^–^* are cheaters. In chimerae, *tagA^–^* mutants also cause wild-type cells to occupy the PST-A region and to be the sole contributor of stalk cells [20], but the *tagA^–^* mutants are not cheaters. This finding suggests that the mechanism of cheating by the *chtC* mutants is more than a passive recognition of a PST-A cell deficiency by the wild-type members of the chimerae.

The mechanism of cheating seen in *chtC* is significantly different from that of *chtA* (*fbxA*) [8]. Though *chtA* is an obligatory cheater that is unable to form spores in clonal populations, it differentiates a higher proportion of prespore cells in slugs [8]. The presence of wild-type cells rescues its development, allowing it to differentiate a higher number of spores in chimeric fruiting bodies. On the other hand, even though the *chtC* mutant has defects in cell-fate maintenance, it is morphologically normal and does not require the presence of wild-type cells to complete development, yet it ends up forming more than its fair share of spores in chimerae with wild-type cells.

Furthermore, while both *chtA* and *chtC* increase the prestalk differentiation of their victims, *chtA* causes the victim's prespore cells to transdifferentiate into stalk cells [8], whereas *chtC* causes a higher number of the victim's cells to initially differentiate as prestalk cells. These observations suggest that *chtC* might be affecting wild-type differentiation earlier than the *chtA* mutant.

### The *chtC* mutants partially overcome the *tagB^–^* prestalk defect

The *tagB^–^* mutant is morphologically rescued when mixed with AX4 cells, and goes on to complete development, although *tagB^–^* cells do not contribute to the PST-A region in the chimerae [14]. However, when mixed with the *chtC* mutants, *tagB^–^* cells become anteriorized and occupy most of the PST-A region, except for the very tip. This finding suggests that the presence of the *chtC* mutants partially overcomes the *tagB^–^* defect. It is therefore likely that the *chtC* mutants affect their chimeric partners before the *tagB* gene acts, in the sequence of developmental events. Since the very tip of the slug does not contain *tagB^–^* prestalk cells (unlike AX4), it is also likely that *tagB^–^* cells are defective in forming several prestalk cell types, and the defect in contributing cells to the very tip of the slug is separate from the PST-A cell defect. The *tagA^–^* mutant, on the other hand, is unaffected by the *chtC* mutants in chimerae, suggesting that the *chtC* gene functions later than *tagA*, and consequently the *chtC* mutants are unable to affect the *tagA^–^* cells. These suggestions are consistent with the timing of expression of the three genes - both *tagA* and *chtC* are expressed throughout development, but their expression peaks at 2 and 12 hours respectively [21]. The *tagB* gene is first induced much later, at about 8 hours, and peaks at 20 hours of development [14].

### A quality-control “check-point” for PST-A cells?

Both the *tagA^–^* and *tagB^–^* mutants have defects in prestalk differentiation, similar to the *chtC* mutants, and both have morphological defects in stalk formation. It has been suggested that the wild type preferentially forms PST-A cells in chimera with these mutants since the mutants are defective in forming those cells [20]. A similar explanation can account for the finding that wild-type cells preferentially contribute to the PST-A region in chimerae with the *chtC* mutants. Though the *chtC* mutants do not appear to be functionally defective in stalk formation, they are defective in the expression of prestalk markers. This observation supports the hypothesis that cells with appropriate expression of prestalk genes contribute preferentially to the stalk (especially the PST-A region), possibly as a form of stalk “quality-control”.

The *chtC* mutants appear to be taking advantage of this PST-A “check-point”. Their presence in chimeric mixtures induces the wild-type cells to form stalk cells even though the *chtC* mutants have the ability to do so themselves, and this leads to an increase in their own spore production at the expense of their victim. This is thus an example of developmental cheating where in the presence of a genetically distinct strain, a cheater mutant is subverting a developmental pathway to increase its own fitness.

Microbial social behaviors are broadly divided into two categories [25] – the production of public goods, and the formation of fruiting bodies as seen in *Dictyostelium* and *Myxococcus xanthus*. While the former is normally concerned with a single (biosynthetic) pathway, the latter may involve various signaling pathways that normally lead to complex developmental processes. Consequently, developmental processes are likely to be manipulated for cheating in these social systems, similar to that seen in super-organisms like social insect colonies. We see an example in this study, where a cheater mutant is manipulating an existing developmental pathway of cell-fate determination and proportioning to exploit other clones. The cooperative system in *Dictyostelium* thus offers a good opportunity to study developmental cheating mechanisms at the genetic and cellular level.

## Materials and Methods

### Strains

The *D. discoideum* strains used in this study are described in Table 2. The *chtC^ins^* strain was described before as the *chtC* mutant [10]. To generate the *chtC^del^* strain, we amplified two fragments from the knockout vector by PCR (Upstream arm primers: 5′-CTTGACATGCGAAATGGC-3′, 5′-GAAGGGACTCCATAAGTATGAG-3′; downstream arm primers: 5′-GTCTTCCAGATGAAAGTTGC-3′, 5′-CCTAATGCAGCACATACTGC-3′). The PCR fragments were cloned between the *Kpn*I and *Cla*I sites of the pLPBLP plasmid, and the entire plasmid was used as a knockout construct to delete most of the endogenous *chtC* gene. For both the *chtC* mutants, the BSR cassette was subsequently removed by transforming the cells with the pDEX-NLS-Cre plasmid [26]. We also created a Cre-expressing plasmid carrying the hygromycin-resistance cassette to use in strains that are already G418-resistant. We transposed the tet^r^-hyg^r^ cassette from the EZTN::tet^r^-A15hyg^r^ plasmid (a kind gift from J. Williams) into the pDEX-NLS-Cre plasmid (just downstream of the *act8* terminator) to generate the pDEX-Cre-hyg^r^ plasmid. The *tagB^–^* mutant was generated by transforming the ptgB-BSR plasmid (a kind gift from W.F. Loomis) into AX4. ptgB-BSR is a *Cla*I-rescued plasmid from a REMI insertion of the pBSRdelBglII plasmid into position 2672 of the *tagB* coding region. The *chtC^ins^* mutation was generated in the AX4/[*cotB*]:*lacZ* (TL1) and AX4/[*ecmA*]:*lacZ* (TL6) strains, and the BSR cassette was subsequently removed by transforming cells with the pDEX-Cre-hyg^r^ plasmid, to give the *chtC^ins^*/[*cotB*]:*lacZ* and *chtC^ins^*/[*ecmA*]:*lacZ* strains respectively. To create the *chtC^del^*/[*cotB*]:*lacZ*, *chtC^del^*/[*ecmA*]:*lacZ*, *chtC^ins^*/[*tagB*]:*lacZ* and *chtC^del^*/[*tagB*]:*lacZ* strains, we transformed the pSP70-LacZ [19], p63NeoGal [27] or the ptagB/lacZ [15] plasmids into the respective *chtC* mutants.

**Table 2:** *D. discoideum* strains used in this study.

Strain Name	Relevant genotype	Parental strain	Drug Marker(s)	Reference
AX4	AX4	AX3		[37]
AX4-GFP	AX4/[*act15*]:*GFP*	AX4	Neo^r^	[38]
TL1	AX4/[*cotB*]:l*lacZ*	AX4	Neo^r^	[19]
TL6	AX4/[*ecmA*]:*lacZ*	AX4	Neo^r^	[18]
	AX4/[*tagB*]:*lacZ*	AX4	Neo^r^	[15]
CCR1	*chtC^ins^*	AX4		This work
CCR2	*chtC^del^*	AX4		This work
CCR3	*chtC^ins^*/[*cotB*]:*lacZ*	TL1	Neo^r^	This work
CCR4	*chtC^ins^*/[*ecmA*]:*lacZ*	TL6	Neo^r^	This work
CCR5	*chtC^ins^*/[*tagB*]:*lacZ*	CCR1	Neo^r^	This work
CCR6	*chtC^del^*/[*cotB*]:*lacZ*	CCR2	Neo^r^	This work
CCR7	*chtC^del^*/[*ecmA*]*lacZ*	CCR2	Neo^r^	This work
CCR8	*chtC^del^*/[*tagB*]:*lacZ*	CCR2	Neo^r^	This work
AK1200	*tagA^–^*	AX4	Bs^r^	[20]
AK1201	*tagA^–^*/[*ecmA*]:*GFP*	TL6	Bs^r^ Neo^r^	[20]
CCR9	*tagB^–^*	AX4	Bs^r^	This work
AK521	*tagB^–^*/[*ecmA*]:*lacZ*	TL51	Neo^r^	[14]

### Growth, transformation

*D. discoideum* cells were grown in suspension cultures in HL5 [28] with the necessary supplements. All strains were grown in HL5 medium without antibiotics for 24–48 hours prior to setting up any experiments, to avoid the potential effects of antibiotics on cell behavior. One labeled strain from each background was mixed with AX4-GFP cells to test the effect of the antibiotic on mixing experiments (Figure S5). Plasmid transformation was carried out essentially as described earlier [29], with the following modifications: cells were resuspended at a final density of 3×10^7^ cells/ml before transformation, electroporated twice, and the transformants were recovered in HL5 with 10% fetal bovine serum for 24 hours prior to the addition of drugs. Depending on the plasmids, transformants were selected with either Blasticidin S (10 µg/ml) or G418 (5 µg/ml). Transformants were grown clonally on SM-agar plates in association with *K. aerogenes* [28], and then re-tested for drug resistance in 24 well-plates containing HL5 with the drug. When appropriate, drug-resistant clones were tested for the correct recombination event by PCR and by Southern blot analysis.

### Development and mixing experiments

We developed cells as described earlier [29] with the following modifications: cells were washed with KK2 buffer (16.3 mM KH_2_PO_4_, 3.7 mM K_2_HPO_4_, pH 6.2), resuspended at a density of 1×10^8^ cells/ml, and 5×10^7^ cells were deposited on each nitrocellulose filter. For the mixes, the cells were grown separately, and mixed before development. We collected all the cells (after 40–48 hours), treated them with 0.1% NP40 to select for spores, and, in the case of GFP-labeled strains, we counted them as described [29]. For mixes with *tagB^–^*, the spores were plated out clonally on SM-agar plates in association with *K. aerogenes* [28], and the plaques were scored by their developmental morphology. For the mixes between the rest of the mutants, spores were plated out similarly, and cells from individual plaques were transferred to two 96-well plates in HL5 containing 10 µg/ml Blasticidin S, and scored for drug-resistance. For the sporulation efficiency experiments, cells were developed as above, and all cells were collected after 40–48 hours of development. NP40-resistance was calculated as the ratio of the number of visible spores after NP40-treatment to the same number prior to NP40-treatment. Sporulation efficiency was calculated as the ratio of the NP40-resistant spores obtained to the number of cells originally plated. These spores were then plated out clonally on SM-agar plates in association with *K. aerogenes* [28] and germination efficiency was calculated as the ratio of the number of plaques obtained to the number of spores plated. For fluorescence microscopy of developing structures with *tagA^–^*/[*ecmA*]:*GFP* cells, the cells were developed on KK2 plates as described [9]. For the segregation assay, we labeled cells with either CellTracker CMFDA or CellTracker Orange CMRA (Molecular Probes) as described [29]. After labeling, we mixed cells from the appropriate strains at a 1∶1 ratio and a final density of 5×10^6^ cells/ml. We then spotted 40 µl of this cell suspension on KK2 (non-nutrient) agar plates, allowed the cells to develop for 8 hours, and then photographed with both transmitted light and fluorescence microscopy. The fluorescence images were overlaid and are shown as color photographs.

### Cell-type specific markers

Developing structures were fixed and stained *in situ* with X-gal (for β-galactosidase activity) as described previously [18], and were counterstained with 0.02% eosin Y [30]. For each experiment, tens of structures were observed in at least 2 independent biological replications, and representative structures are shown in the figures. Staining of dissociated cells was done essentially as described earlier [18], except that the developing structures were passed through an 18G1½ needle, and treated with pronase (0.1% pronase, 0.1% β-mercaptoethanol, 150 mM NaCl, 50 mM Tris pH 7.0) for 10 minutes at room temperature for efficient dissociation. GFP-labeled cells were counted directly after dissociation using phase-contrast and fluorescence microscopy. For slug migration, cells were washed twice with double-distilled water, and 10^8^ cells were streaked on 2% Agar-Noble plates made with double-distilled water. The plates were incubated in a dark chamber with a unidirectional source of light for 48 hours, and exposed to overhead light to induce culmination. Developing structures were collected after migration and stained as above. Spore staining was carried out as described previously [18].

### Nucleic acid analysis

Genomic DNA was prepared as described earlier by the CTAB method [31]. Southern blot analysis was performed by standard methods [32]. RNA extraction and Northern blot analysis were performed as described previously [33]. The blots were hybridized with DNA probes made by random-primer labeling [34]. We used a PCR fragment from pLAS5 [9] to probe for the *chtC* gene. The abundance of the *chtC* mRNA was determined by Q-RT-PCR as described, using *rnlA* (*Ig7*) to normalize for cDNA levels [35]. The primers used were: *chtC* 5′-TTCACCAAATCCACTAGACTGTC-3′ and 5′-CAGTTGCTTTCTTACGTGCAAG-3′and *Ig7* 5′-TTACATTTATTAGACCCG AAACCAAGC-3′ and 5′-TTCCCTTTAGACCTATGGACCTTAGCG-3′. The abundance of the *tagB* mRNA was also similarly determined by Q-RT-PCR (primers: 5′-TTTCCCAACTGGCGAATC-3′ and 5′-CCTAAACCACCGATACCAATC-3′). *In situ* RNA hybridization was done as described [36] with the following modifications: hybridization was done in the same solution as the pre-hybridization; both steps, as well as washing were done at 50°C, and the final wash was done in 0.1X SSC. A digoxigenin-labeled RNA probe was made by *in vitro* transcription from the plasmid pLAS5 using the T7 promoter with the DIG RNA labeling kit from Roche.

## Supporting Information

Figure S1 The *chtC* mutants do not exhibit sporulation defects. AX4, *chtC^ins^* and *chtC^ del^* cells were grown clonally and then mixed before development (where indicated) for 40–48 hours. Spores were collected, and the detergent-resistance of the spores, sporulation efficiency, and germination efficiency of the samples were determined. The AX4 values were normalized to 100% (the sporulation efficiency of AX4 was 134.4%±14.9%), and all the values are presented relative to AX4, and are shown as the means and standard errors of three independent replications. None of the samples were significantly different from AX4 (*P*>0.1, Student's *t*-test).
(0.13 MB TIF)

Figure S2 The *chtC* mutants co-aggregate with wild-type cells. Strains were grown clonally, labeled with a CellTracker dye, and then mixed before development. AX4, *chtC^ins^* and *chtC^del^* cells labeled with CellTracker Orange CMRA were mixed at a 1∶1 ratio with AX4 cells labeled with CellTracker Green CMFDA and photographed after 8 hours of development. Both the *chtC* mutants co-aggregate with wild-type cells, similar to the AX4 control. The scale bar represents 0.1 mm.
(3.20 MB TIF)

Figure S3 *tagB* expression in prespore cells is not maintained till late development in the *chtC* mutants. Quantitative reverse-transcriptase PCR with primers specific to *tagB* performed on RNA samples collected from AX4, *chtC^ins^* and *chtC^del^*strains at 16 h of development, and from spores. Data are presented as the fold change relative to the level in AX4 spores (y-axis) and are the averages and standard errors of 3 measurements each of at least 2 independent biological replications. The expression levels in the spores of the *chtC* mutants are not higher than those in AX4 (Student's *t*-test).
(0.05 MB TIF)

Figure S4 The *chtC* mutants do not show prespore to prestalk transdifferentiation. AX4, *chtC^ins^* and *chtC^del^* strains labeled with [*cotB*]:*lacZ* were developed for 16 hours, fixed and stained with X-gal. In both cases, 10% of the cells were labeled and the remaining population consisted of the unlabeled parental strain. Representative slugs for each strain are shown. The scale bars represent 0.1 mm.
(0.75 MB TIF)

Figure S5 Labeled strains are similar to their unlabeled parents in mixes with wild-type cells. Strains were grown clonally and then mixed before development. One labeled strain from each parental background was mixed in a 1∶1 ratio with AX4-GFP cells, and their spore production was measured. Data are presented as the proportion (%) of the spores produced by the strain of interest relative to the total spores produced by the chimerae. The results are the means and standard errors of at least 3 independent replications. Only the *chtC* mutants form significantly different proportions of spores compared to the AX4 control (Student's *t*-test). The *P*-values for each strain (corrected for multiple testing using the ‘Benjamini and Hochberg’ method) are shown below the respective bars. None of the labeled strains are significantly different from their unlabeled parental strains in similar mixes (Student's *t*-test).
(0.05 MB TIF)
