# Supplementary figures and images for: Cheating by Exploitation of Developmental Prestalk Patterning in Dictyostelium discoideum
Source: PLoS Genet. 2010 Feb 26;6(2):e1000854. doi: 10.1371/journal.pgen.1000854 (PMC2829058; doi:10.1371/journal.pgen.1000854)

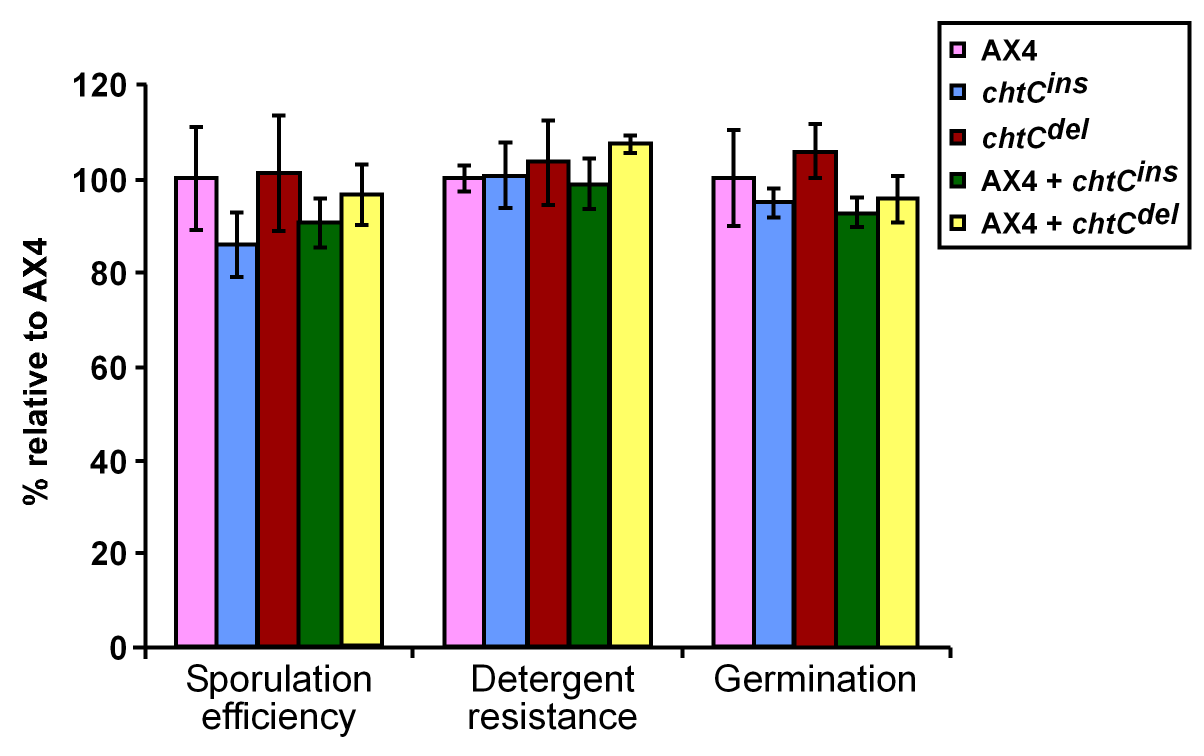

Supplement: Figure S1 — The chtC mutants do not exhibit sporulation defects. AX4, chtCins and chtC del cells were grown clonally and then mixed before development (where indicated) for 40–48 hours. Spores were collected, and the detergent-resistance of the spores, sporulation efficiency, and germination efficiency of the samples were determined. The AX4 values were normalized to 100% (the sporulation efficiency of AX4 was 134.4%±14.9%), and all the values are presented relative to AX4, and are shown as the means and standard errors of three independent replications. None of the samples were significantly different from AX4 (P>0.1, Student's t-test). (0.13 MB TIF) [file pgen.1000854.s001.tif]

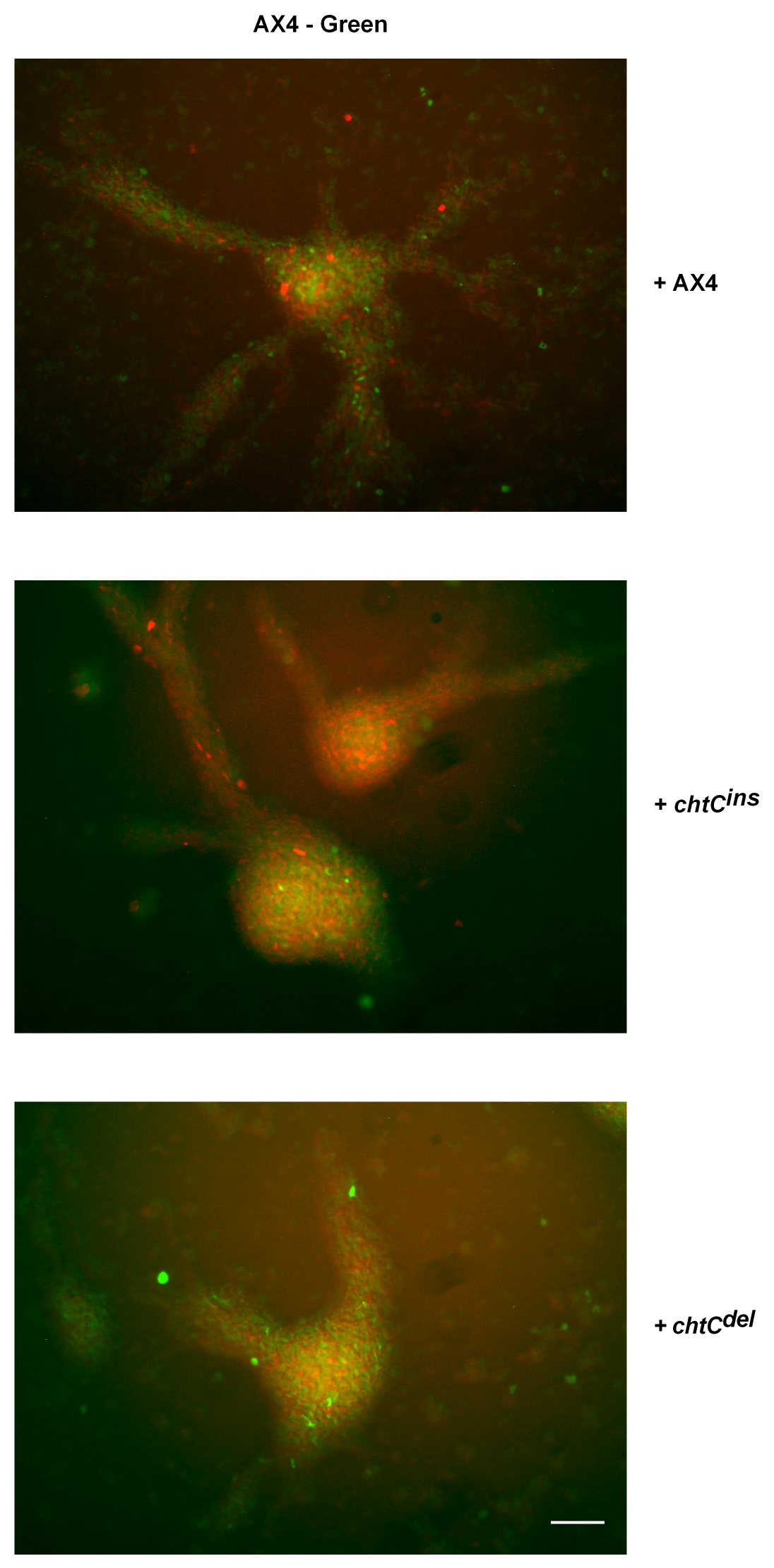

Supplement: Figure S2 — The chtC mutants co-aggregate with wild-type cells. Strains were grown clonally, labeled with a CellTracker dye, and then mixed before development. AX4, chtCins and chtCdel cells labeled with CellTracker Orange CMRA were mixed at a 1∶1 ratio with AX4 cells labeled with CellTracker Green CMFDA and photographed after 8 hours of development. Both the chtC mutants co-aggregate with wild-type cells, similar to the AX4 control. The scale bar represents 0.1 mm. (3.20 MB TIF) [file pgen.1000854.s002.tif]

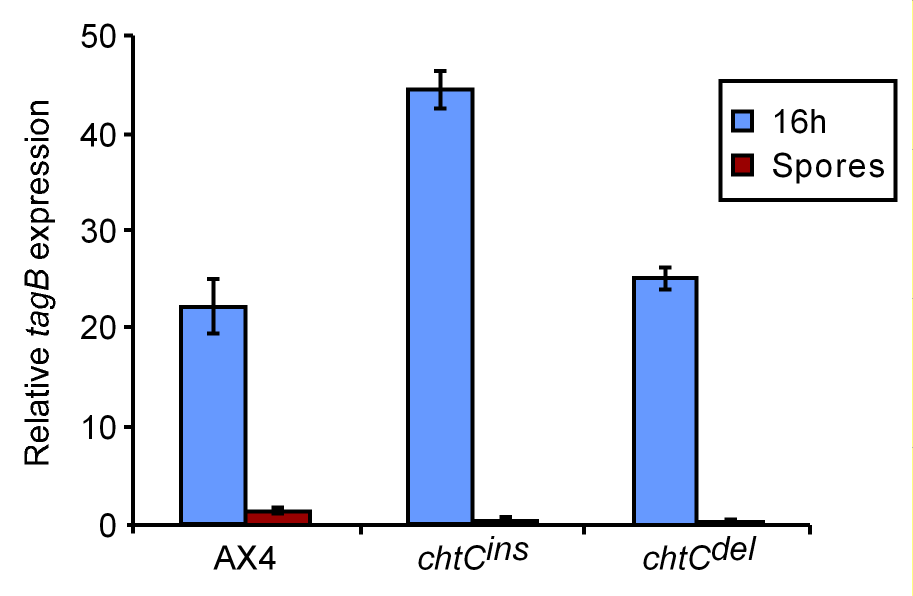

Supplement: Figure S3 — tagB expression in prespore cells is not maintained till late development in the chtC mutants. Quantitative reverse-transcriptase PCR with primers specific to tagB performed on RNA samples collected from AX4, chtCins and chtCdelstrains at 16 h of development, and from spores. Data are presented as the fold change relative to the level in AX4 spores (y-axis) and are the averages and standard errors of 3 measurements each of at least 2 independent biological replications. The expression levels in the spores of the chtC mutants are not higher than those in AX4 (Student's t-test). (0.05 MB TIF) [file pgen.1000854.s003.tif]

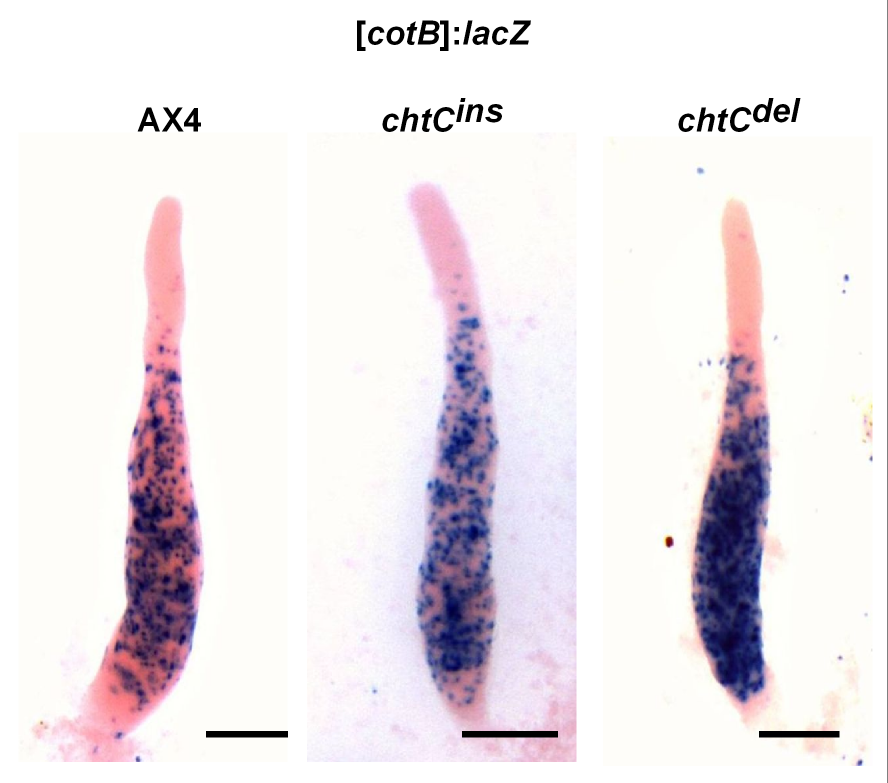

Supplement: Figure S4 — The chtC mutants do not show prespore to prestalk transdifferentiation. AX4, chtCins and chtCdel strains labeled with [cotB]:lacZ were developed for 16 hours, fixed and stained with X-gal. In both cases, 10% of the cells were labeled and the remaining population consisted of the unlabeled parental strain. Representative slugs for each strain are shown. The scale bars represent 0.1 mm. (0.75 MB TIF) [file pgen.1000854.s004.tif]

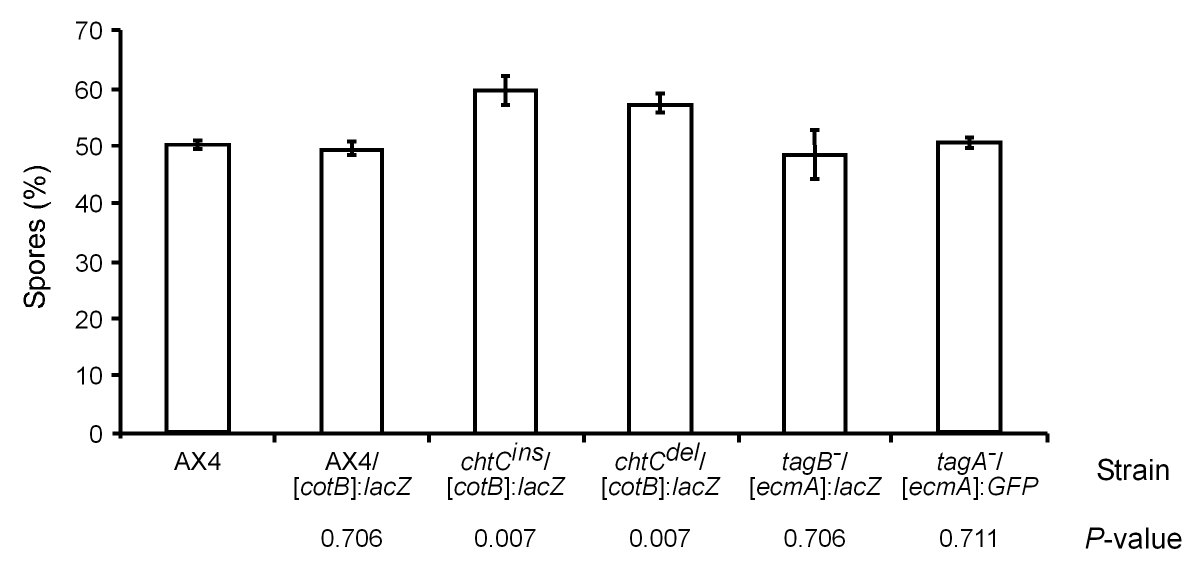

Supplement: Figure S5 — Labeled strains are similar to their unlabeled parents in mixes with wild-type cells. Strains were grown clonally and then mixed before development. One labeled strain from each parental background was mixed in a 1∶1 ratio with AX4-GFP cells, and their spore production was measured. Data are presented as the proportion (%) of the spores produced by the strain of interest relative to the total spores produced by the chimerae. The results are the means and standard errors of at least 3 independent replications. Only the chtC mutants form significantly different proportions of spores compared to the AX4 control (Student's t-test). The P-values for each strain (corrected for multiple testing using the ‘Benjamini and Hochberg’ method) are shown below the respective bars. None of the labeled strains are significantly different from their unlabeled parental strains in similar mixes (Student's t-test). (0.05 MB TIF) [file pgen.1000854.s005.tif]
